# MntR Mediates LiaSR-Regulated *gadT2*/*gadD2* Expression and Acid Resistance in *Listeria monocytogenes* 10403S

**DOI:** 10.3390/microorganisms14081765

**Published:** 2026-08-11

**Authors:** Yuhang Yang, Minghao Zheng, Xu Han, Jinhua Xiao, Xiongyan Liang, Jing Liu, Lei Tan, Yuying Yang, Shouguo Fang, Xiaowei Fang, Chun Fang

**Affiliations:** 1 College of Animal Science and Technology, Yangtze University, No. 88, Jingmi Road, Jingzhou 434025, China; 2 Department of Animal Science, Jiangxi Biotech Vocational College, No. 1636, Liantang North Road, Nanchang 330200, China; 3 College of Agriculture, Yangtze University, No. 88, Jingmi Road, Jingzhou 434025, China

**Keywords:** *Listeria monocytogenes*, two-component system, transcription factor, glutamate decarboxylase, acid resistance

## Abstract

*Listeria monocytogenes* is a foodborne pathogen capable of persisting under acid, osmotic, oxidative, thermal, and other environmental stresses. The glutamate decarboxylase (GAD) system is a major determinant of survival under acidic conditions. Previous work showed that the two-component system LiaSR negatively regulates the *gadT2*/*gadD2* locus in *L. monocytogenes* 10403S, but the intervening regulator was unknown. Here, we combined transcription-factor mutant screening, promoter-reporter assays, RT-qPCR, Western blotting, acid-survival assays, and electrophoretic mobility shift assays to define this regulatory relationship. Deletion of *mntR* reduced P*_gadT2_*-*gfp* activity, *gadD2* transcript abundance, and GadD2 protein levels at pH 4.5, 7, and 9, whereas complementation restored expression toward the wild-type level. The Δ*mntR* strain also showed reduced survival under inorganic and organic acid stresses. LiaSR deletion increased P*_mntR_*-*gfp* activity, and LiaR bound directly to P*_mntR_*. Conversely, *mntR* deletion increased LiaR abundance, although MntR did not bind to P*_liaSR_*, indicating indirect feedback regulation. The *liaSR*/*mntR* double mutant showed reduced *gadT2*/*gadD2* expression under acidic conditions and impaired acid survival. Together, these findings support a LiaSR-MntR-*gadT2*/*gadD2* pathway in which MntR promotes GAD-mediated acid resistance.

## 1. Introduction

*L. monocytogenes* is a Gram-positive bacterium that infects humans and animals through contaminated food, causing listeriosis [1]. Although listeriosis is relatively uncommon, it is associated with high rates of hospitalization and death. In 2024, 26 European Union Member States reported 3041 confirmed cases of invasive listeriosis, corresponding to a notification rate of 0.69 cases per 100,000 population. Of the cases with available information, 97.3% required hospitalization, and 301 deaths were reported, 81.7% of which occurred in individuals older than 65 years. Both the number of cases and the notification rate were the highest recorded since EU-level surveillance began in 2007, with a significant increasing trend during 2020–2024 [2]. In China, sentinel surveillance in Beijing identified 228 invasive listeriosis cases during 2013–2022, with a mean annual morbidity of 1.054 cases per million inhabitants and an overall case-fatality rate of 31.82%. Maternal–neonatal listeriosis occurred at 63.401 cases per million live births and had a case-fatality rate of 42.86% [3]. Recent outbreak investigations also demonstrate the ability of *L. monocytogenes* to persist in food-production environments. Two outbreaks associated with packaged salads caused 30 illnesses, 27 hospitalizations, and four deaths in the United States and Canada; illnesses linked to the two outbreak strains occurred over periods of five to eight years [4]. *L. monocytogenes* is widely distributed in nature and can be found in soil, water sources and various foods, posing a great threat to the food industry [5]. This bacterium can adapt to harsh environments, especially acidic environments [6]. It can also tolerate refrigeration, osmotic, oxidative, thermal, and disinfectant stresses [3,7,8]. During food processing, passage through the host gastrointestinal tract or survival in the phagosome, *L. monocytogenes* will encounter acid stress, and resistance to acid stress is important for successful infection [9].

The GAD system is a common acid-resistance system in bacteria, which is usually composed of membrane glutamate transporters and cytoplasmic glutamate decarboxylases [10]. The acid resistance of the GAD system is achieved by the decarboxylation of glutamate, which consumes 1 mol of glutamate salt and 1 mol of H^+^, and produces 1 mol of γ-aminobutyric acid (GABA), 1 mol of H_2_O and 1 mol of CO_2_ [11]. This reaction consumes H^+^ and helps raise the intracellular pH. There are usually 2–3 groups of glutamate transporter–glutamate decarboxylase (*gadT*-*gadD*) in *L. monocytogenes*, and each component plays different roles in the acid resistance of *L. monocytogenes* [12]. Studies have shown that *gadT1*/*gadD1* plays a role in acid resistance in weak acid environments, *gadD3* plays a major role in acid resistance in the EGD-e strain, and *gadT2*/*gadD2* is the key factor for acid resistance in strong acid environments [13,14,15]. The present study focuses on the regulatory mechanism of the GAD system. Previous studies have shown that GadT1/GadD1 and GadD3 are regulated by the stress regulatory factor SigB [13,16,17], but the regulation of *gadT2*/*gadD2*, the major contributor to acid resistance, is unclear.

Bacteria use signal transduction systems to respond to external environments, and the two-component system (TCS) is one important group [18]. The classical TCS consists of a histidine kinase (HK) located on the cell membrane and a response regulator (RR) located in the cytoplasm. HK senses environmental signals, undergoes auto-phosphorylation on its histidine residue, and transfers the phosphate group to RR. RR undergoes phosphorylation on its aspartate residue, and binds downstream target genes and regulates transcription [19]. In *L. monocytogenes*, two-component systems are involved in regulating bacterial antibiotic tolerance, biofilm formation, virulence and stress resistance [7,8,20,21]. Among them, the two-component system LiaSR has been reported to regulate the resistance to antibiotics targeting the cell membrane, as well as the tolerance to heat, acid, alkali, osmotic, ethanol and oxidative stresses [21,22]. We previously screened the two-component systems of *L. monocytogenes* 10403S and found that the deletion of LiaSR significantly increased the expression of *gadT2*/*gadD2* [23], but the mechanism was unclear. Transcription regulation in bacteria is usually accomplished by transcription factors, which bind to the promoter regions of their target genes to achieve transcription regulation [24]. Transcription factors act on various processes of bacterial life, including bacterial resistance to environmental stress [25]. In *L. monocytogenes*, transcription factors have been reported to be involved in regulating cold, acid, osmotic, ethanol, and other stresses [25,26,27,28,29]. We previously reported that the transcription factor GadR4 participates in regulating the acid resistance of *L. monocytogenes* 10403S and affects the expression of *gadT2*/*gadD2* [15]. However, whether transcription factors are involved in LiaSR-mediated regulation of *gadT2*/*gadD2* remains unclear.

This study aimed to identify the transcription factor that regulates *gadT2*/*gadD2*, and to examine how LiaSR regulates this transcription factor and thereby regulates *gadT2*/*gadD2*.

## 2. Materials and Methods

### 2.1. Plasmids, Strains and Culture Conditions

The wild-type strain 10403S used in this study was obtained from our previous work [15]. The vectors pKSV7, pERL3 and pIMK2 were maintained in *E. coli* DH5α. *E. coli* was cultured in Luria–Bertani broth (LB, Oxoid, McHenry, IL, USA), and *L. monocytogenes* was cultured in brain–heart infusion broth (BHI, Oxoid, McHenry, IL, USA).

### 2.2. Gene Deletion and Complementation

Gene deletion and complementation mutants were constructed according to our previous work [15]. Briefly, 600 bp sequences upstream and downstream of the target gene were selected as homologous arms, and primers for amplifying the homologous arms were designed (Table S1). Using the *L. monocytogenes* 10403S genome as a template, the homologous arms were amplified and fused by PCR. The fused homologous arms were cloned into the pKSV7 vector and electroporated into *L. monocytogenes* 10403S cells. The successfully electroporated *L. monocytogenes* was continuously passaged at 42 °C to achieve homologous recombination, and the successfully recombined *L. monocytogenes* was continuously passaged at 28 °C to eliminate the plasmid, resulting in the gene deletion mutant.

For complementation, the target gene was cloned into the pIMK2 vector and transferred into the gene deletion mutant by electroporation, resulting in the complementation mutant.

### 2.3. Construction of Gfp Reporter Plasmid and Fluorescence Value Analysis

The *gfp* reporter plasmid was constructed according to our previous report [30]. Primers for the target gene promoter region and *gfp* were designed (Table S1), and the target gene promoter and *gfp* fragments were amplified by PCR. The promoter and *gfp* fragments were fused by overlap extension PCR, and the fused fragment was cloned into the pERL3 vector. The recombinant plasmid was then electroporated into *L. monocytogenes* wild type, gene deletion mutant and gene complementation mutant to obtain strains carrying the *gfp* reporter plasmid.

The *gfp* reporter strains were inoculated in BHI medium and cultured at 37 °C for 16 h. The next day, cells were collected by centrifugation at 12,000 rpm for 2 min; resuspended in 10 mM phosphate-buffered saline (PBS) (Servicebio, G4207, Wuhan, China) with pH 4.5, pH 7 or pH 9; and treated at 37 °C for 1 h. After the stress treatment, the cells were resuspended in PBS and added to a 96-well microplate, at 200 μL per well. Fluorescence values were measured at excitation and emission wavelengths of 485 nm and 535 nm, respectively, by Sense Microplate Reader (HIDEX, Turku, Finland), once per hour to generate fluorescence curves.

### 2.4. Growth Curve Determination

Growth curve determination was performed according to a previously reported method [15]. *L. monocytogenes* was inoculated in BHI medium and cultured at 37 °C for 16 h. The next day, cells were collected by centrifugation at 12,000 rpm for 2 min and resuspended in PBS, and added to a 96-well microplate, at 200 μL per well. The OD_600nm_ values were measured by Sense Microplate Reader (HIDEX, Turku, Finland), once per hour for 14 h at 37 °C.

### 2.5. Western Blot Analysis

Western blot analysis was performed according to our previous report [30]. Overnight-cultured *L. monocytogenes* was collected by centrifugation at 12,000 rpm for 2 min; resuspended in PBS with pH 4.5, pH 7 or pH 9; and treated at 37 °C for 3 h. Cells were collected by centrifugation, resuspended in bacterial lysis buffer (1% SDS (Beyotime, ST627, Shanghai, China), 2% Triton X-100 (Beyotime, ST1723-100mL, Shanghai, China)), and subjected to repeated freezing and boiling cycles. The supernatant was collected by centrifugation, and the supernatant was used as the protein sample. The target protein was resolved by 12% SDS-PAGE and blotted onto a PVDF membrane (Merck Millipore, Darmstadt, Germany). The membrane was incubated with 5% skim milk for 2 h and then with a specific antibody against the target protein at 4 °C overnight. The next day, the membrane was washed thoroughly and incubated with HRP-labeled goat anti-rabbit antibody (Sangon Biotech, NO. D110058, Shanghai, China) for 1 h, and then washed again and exposed to a chemiluminescence imaging system (G-box, Syngene, Cambridge, UK). The band intensities were analyzed using ImageJ software (ImageJ 1.52V, National Institutes of Health, Bethesda, MD, USA).

### 2.6. Reverse-Transcription Quantitative PCR

The acid stress survival assay was performed according to a previous report [15]. Strains 10403S, Δ*mntR*, CΔ*mntR*, and Δ*liaSR*/*mntR* were cultured and exposed to pH 4.5, 7, or 9 at 37 °C for 3 h, as described for Western blotting. Cells were collected immediately after treatment. Total RNA was extracted using Total RNA Extraction Reagent (TRIzol; ABclonal Technology Co., Ltd., Cat. No. RK30129, Wuhan, China) according to the manufacturer’s protocol. Residual genomic DNA was removed using DNase I, RNase-free (5000 U/mL; ABclonal Technology Co., Ltd., Cat. No. RK20549, Wuhan, China). RNA concentration and purity were determined spectrophotometrically, and integrity was evaluated by agarose gel electrophoresis. One microgram of total RNA was reverse-transcribed in a 20-μL reaction using ABScript III RT Master Mix for qPCR (ABclonal Technology Co., Ltd., Cat. No. RK20428, Wuhan, China) at 55 °C for 15 min, followed by 85 °C for 5 min. RT-qPCR was performed in 20-μL reactions containing 10 μL of 2X Universal SYBR Green Fast qPCR Mix (ABclonal Technology Co., Ltd., Cat. No. RK21203, Wuhan, China), 0.4 μL each of 10 μM forward and reverse primers, 2 μL of cDNA, and nuclease-free water. Amplification was conducted using a QuantStudio 5 Real-Time PCR System (Applied Biosystems, Thermo Fisher Scientific, Waltham, MA, USA) with an initial denaturation at 95 °C for 3 min, followed by 40 cycles of 95 °C for 5 s and 60 °C for 30 s. A melting-curve analysis was performed after amplification, and no-template and no-reverse-transcription controls were included. *gyrB* was used for normalization, and relative *gadD2* transcript abundance was calculated using the 2^−ΔΔCt^ method, with 10403S under each pH condition set to 1. Three independent biological replicates were analyzed, each with three technical replicates. Primer sequences are listed in Table S1.

### 2.7. Acid Stress Survival Assay

The acid stress survival assay was performed according to the protocol we previously reported [15]. Overnight-cultured *L. monocytogenes* was collected by centrifugation and resuspended in PBS adjusted to pH 2.5 with 10 M HCl (Sigma-Aldrich, 258148, Darmstadt, Germany), BHI containing 2% (*v*/*v*) lactic acid (Sigma-Aldrich, L6661, Darmstadt, Germany), BHI containing 2% (*v*/*v*) acetic acid (Sigma-Aldrich, 695092, Darmstadt, Germany), and BHI containing 2% (*w*/*v*) citric acid (Sigma-Aldrich, 251275, Darmstadt, Germany) and treated at 37 °C for acid stress. The 2% lactic acid treatment was carried out for 0.5 h, while the pH 2.5, 2% acetic acid, and 2% citric acid treatments were carried out for 1 h each. At the same time, the bacteria before the treatment were diluted 10-fold and plated for counting, and the bacterial number obtained was recorded as the initial amount F1. After the acid stress treatment, the bacteria were also diluted 10-fold and plated for counting, and the bacterial number obtained was recorded as the survival amount F2. The final survival rate was calculated as F2/F1 × 100%.

### 2.8. Electrophoretic Mobility Shift Assay

Electrophoretic mobility shift assay (EMSA) was performed according to a previous report [31]. Different concentrations of protein were mixed with 200 ng of promoter DNA and Binding Buffer (50 mM Tris-HCl (Sigma-Aldrich, 108315, Darmstadt, Germany) (pH 8), 250 mM NaCl (Sigma-Aldrich, S9888, Darmstadt, Germany), 5 mM MgCl_2_ (Sigma-Aldrich, 208337, Darmstadt, Germany), 2.5 mM DTT (Sigma-Aldrich, DTT-RO, Darmstadt, Germany), 2.5 mM EDTA (Sigma-Aldrich, E9884, Darmstadt, Germany), and 20% glycerol (Sigma-Aldrich, G5516, Darmstadt, Germany)), with a total volume of 20 μL. The mixture was incubated at 37 °C for 30 min, and then separated by 4% native PAGE. Finally, the PAGE was stained with nucleic acid dye (Sangon Biotech, NO. A616697, Shanghai, China) for 15 min, and DNA–protein complexes were observed under UV irradiation.

### 2.9. Statistical Analysis

Statistical analyses were performed using GraphPad Prism 6 (GraphPad Software, Boston, MA, USA). For comparisons among two strains under a single condition, unpaired *t*-test was used. For comparisons among three strains under a single condition, one-way ANOVA followed by Tukey’s multiple-comparison test was used. Results are presented as mean ± SD of three independent experiments, and *p* < 0.05 was considered statistically significant. * *p* < 0.05; ** *p* < 0.01; and ns indicates non-significant differences (*p* > 0.05).

## 3. Results

### 3.1. Deletion of mntR Has the Greatest Impact on gadT2/gadD2 Expression

**Figure 1:**
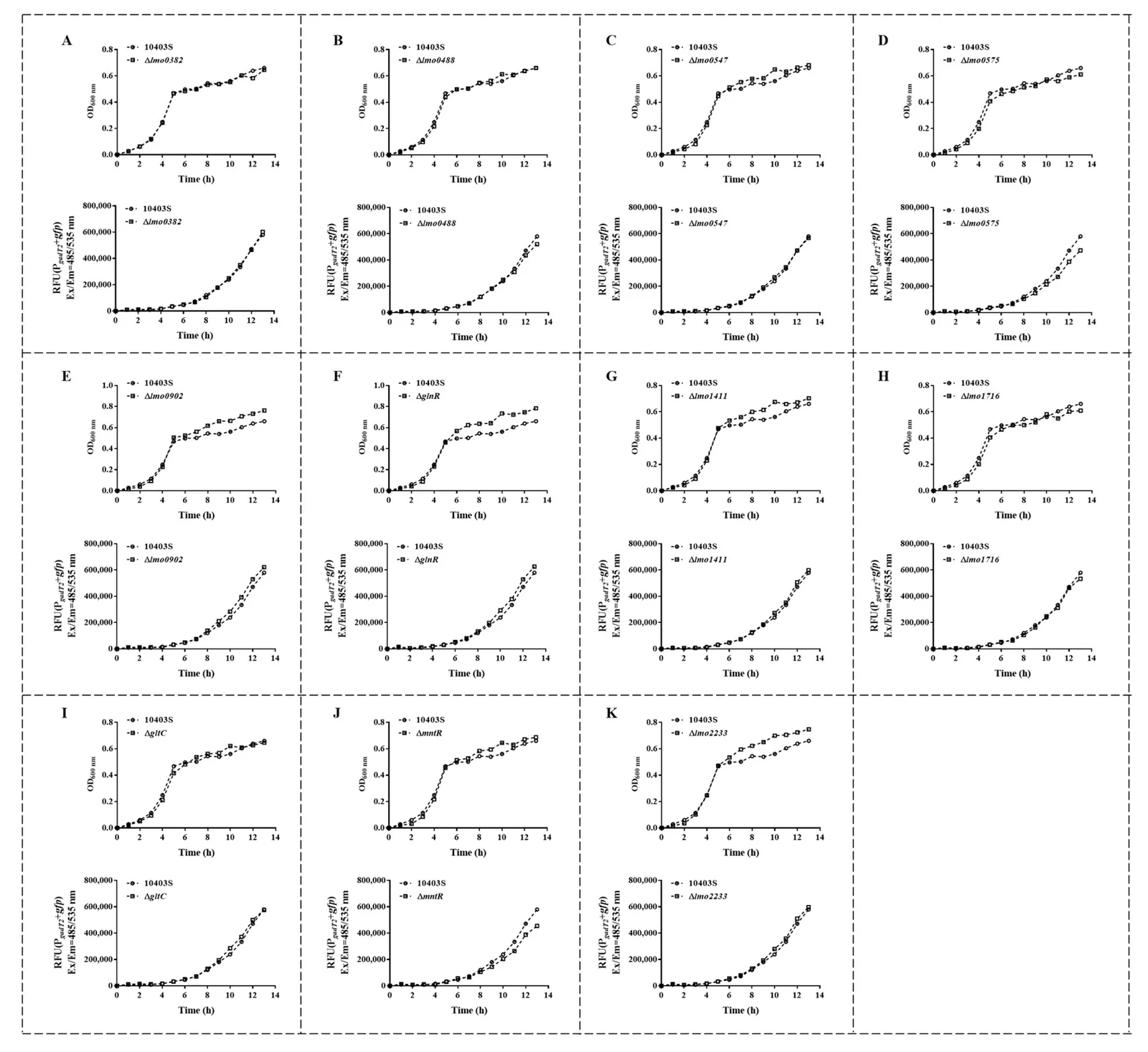
Growth curves and fluorescence curves of transcription factor deletion strains. (**A**) Δ*lmo0382*. (**B**) Δ*lmo0488*. (**C**) Δ*lmo0547*. (**D**) Δ*lmo0575*. (**E**) Δ*lmo0902*. (**F**) Δ*glnR*. (**G**) Δ*lmo1411*. (**H**) Δ*lmo1716*. (**I**) Δ*gltC*. (**J**) Δ*mntR*. (**K**) Δ*lmo2233*. The mean ± SD of three independent experiments is shown in the data.

To screen for transcription factors that regulate *gadT2*/*gadD2*, 11 transcription factor deletion mutants were constructed, and *gadT2*/*gadD2* expression was compared between the deletion strains and the wild type (Figure 1 and Figure S1). The fluorescence curve results showed that after 8 h, the fluorescence value of Δ*mntR* was lower than that of 10403S. Over time, the difference between the two gradually increased, and the OD_600nm_ value of Δ*mntR* was higher than that of 10403S. The bacterial count of Δ*mntR* was higher than that of 10403S, but the fluorescence value was lower (Figure 1J), indicating that the deletion of *mntR* significantly reduced the expression of *gadT2*/*gadD2*. The fluorescence curves of other transcription factor deletion mutants showed no significant difference compared to the wild type (Figure 1). These fluorescence curves identified MntR as a transcription factor that positively regulates the expression of *gadT2*/*gadD2*.

### 3.2. MntR Positively Regulates the Expression of gadT2/gadD2 by Directly Binding to P_gadT2_

**Figure 2:**
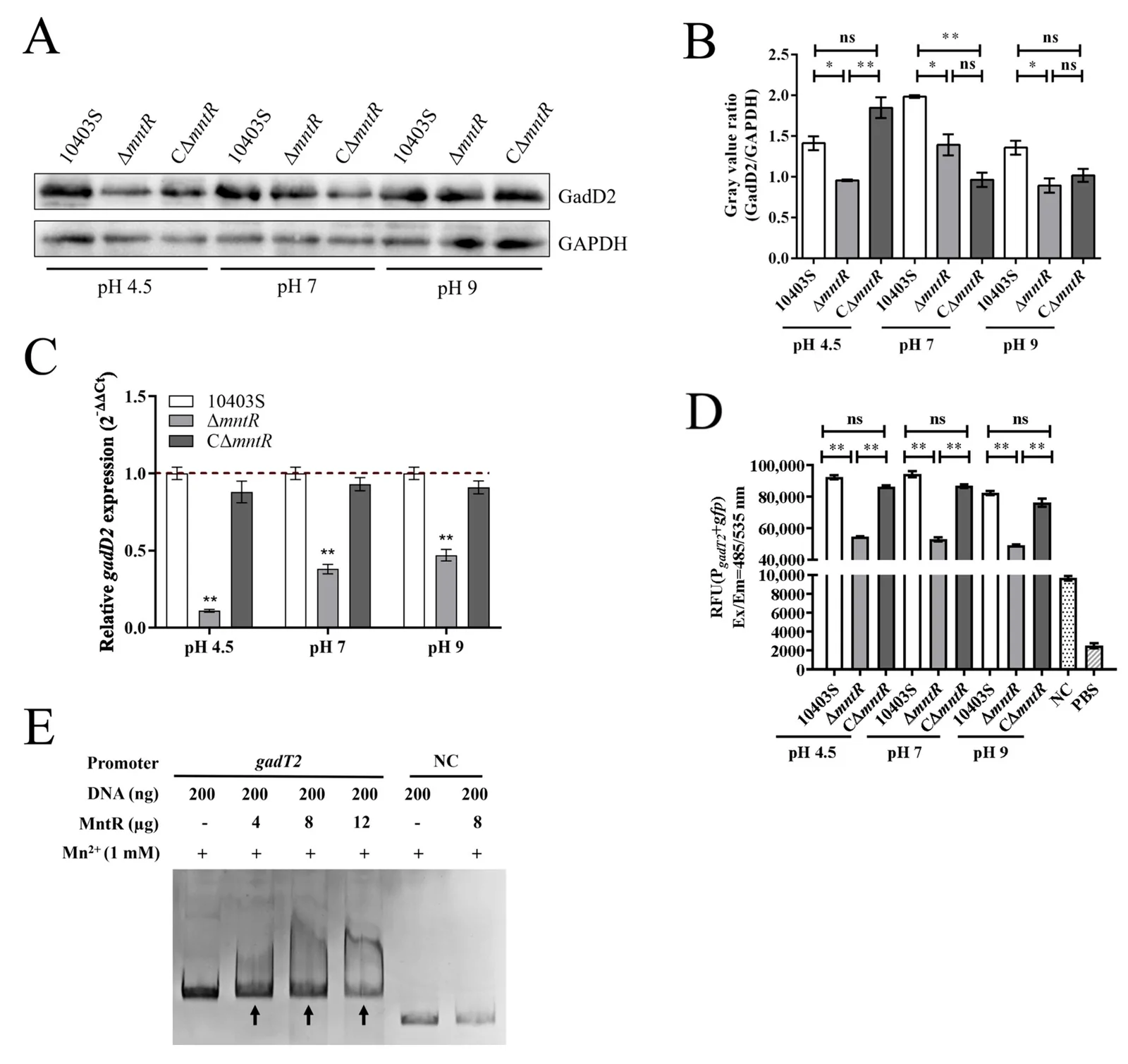
The regulation of *gadT2*/*gadD2* by MntR. (**A**) Expression of GadD2 in 10403S, Δ*mntR*, and CΔ*mntR* under pH 4.5, pH 7, and pH 9 conditions. (**B**) Grayscale analysis of GadD2 expression. (**C**) Relative *gadD2* transcript abundance determined by RT-qPCR. The expression level of 10403S under each pH condition was set to 1. (**D**) Expression of P*_gadT2_*-*gfp* in 10403S, Δ*mntR*, and CΔ*mntR* under pH 4.5, pH 7, and pH 9 conditions. (**E**) The electrophoretic mobility shift assay (EMSA) of MntR and P*_gadT2_*, the retarded band (arrows) indicates the DNA–protein complex. Data are presented as the mean ± SD from three independent biological replicates. ns: *p* > 0.05; *: *p* < 0.05; **: *p* < 0.01.

Next, the regulatory effect of MntR on *gadT2*/*gadD2* expression was examined under different pH conditions. Fluorescence results showed that the fluorescence values of Δ*mntR* were significantly lower than those of 10403S at pH 4.5, pH 7 or pH 9 (Figure 2D), and there was no significant difference between CΔ*mntR* and 10403S, indicating that *mntR* deletion significantly reduced the expression of *gadT2*/*gadD2*. Western blot results showed that at pH 4.5, pH 7 and pH 9, the expression of GadD2 in Δ*mntR* was significantly lower than that of 10403S (Figure 2A,B), indicating that *mntR* deletion significantly decreased the expression of *gadT2*/*gadD2* in acidic, neutral or alkaline environments. RT-qPCR further showed that *gadD2* transcript abundance was significantly reduced in Δ*mntR* under pH 4.5, pH 7, and pH 9 conditions, with the largest reduction at pH 4.5, whereas CΔ*mntR* showed expression close to that of 10403S (Figure 2C). EMSA results showed that a mobility shift was observed after adding 12 μg, 8 μg, and 4 μg of MntR protein, respectively (Figure 2E), indicating that MntR directly bound to P*_gadT2_*.

### 3.3. MntR Positively Regulates the Acid Resistance of L. monocytogenes 10403S

**Figure 3:**
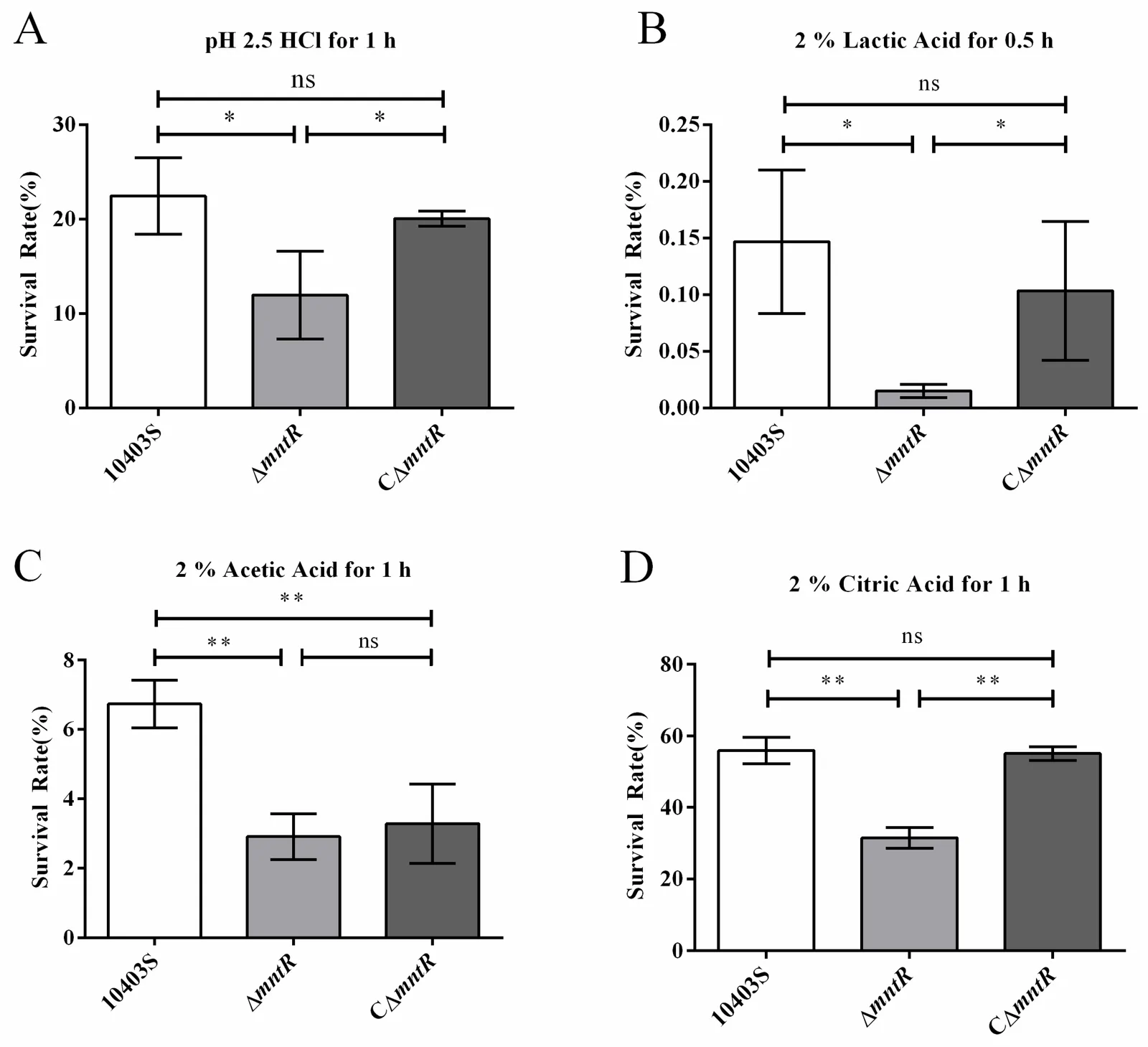
Survival assays of 10403S, Δ*mntR*, and CΔ*mntR* under various acidic conditions: (**A**) pH 2.5 HCl for 1 h, (**B**) 2% lactic acid for 0.5 h, (**C**) 2% acetic acid for 1 h, (**D**) 2% citric acid for 1 h. The mean ± SD of three independent experiments is shown in the data. ns: *p* > 0.05; *: *p* < 0.05; **: *p* < 0.01.

Survival was also compared among 10403S, Δ*mntR*, and CΔ*mntR* after treatment with inorganic and organic acids. The results showed that after treatment with pH 2.5 PBS for 1 h, with BHI containing 2% lactic acid for 0.5 h, or with BHI containing 2% citric acid for 1 h, the survival rate of Δ*mntR* was significantly lower than that of 10403S and CΔ*mntR,* and there was no significant difference between CΔ*mntR* and 10403S (Figure 3A,B,D). After treatment with BHI containing 2% acetic acid for 1 h, the survival rate of Δ*mntR* was significantly lower than that of 10403S (Figure 3C). Together, the acid stress survival assays showed that the deletion of *mntR* significantly weakened the acid resistance of *L. monocytogenes* 10403S.

### 3.4. LiaSR Negatively Regulates the Expression of MntR by Directly Binding to P_mntR_

**Figure 4:**
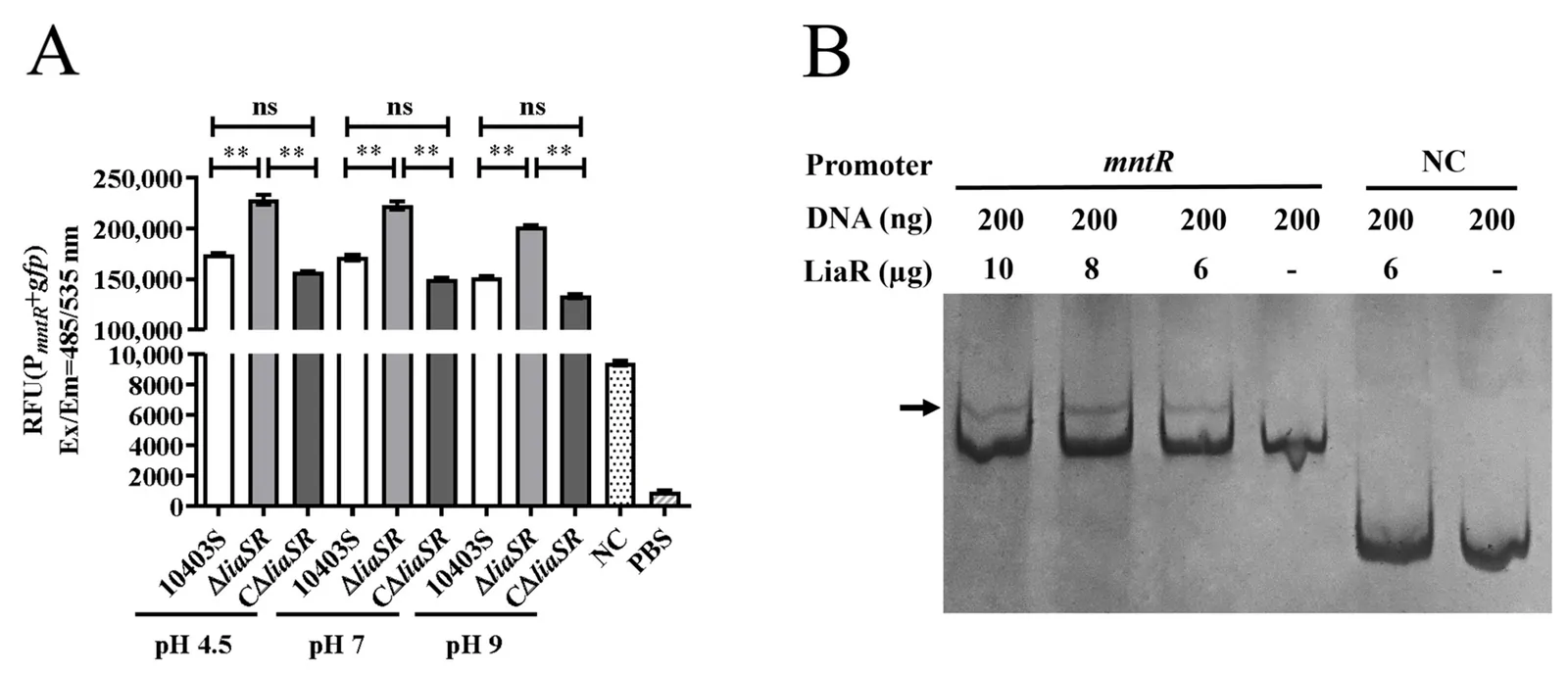
The regulation of MntR by LiaSR. (**A**) Expression of P*_mntR_*-*gfp* in 10403S, Δ*liaSR*, and CΔ*liaSR* under pH 4.5, pH 7, and pH 9 conditions. (**B**) The electrophoretic mobility shift assay (EMSA) of LiaR and P*_mntR_*, the retarded band (arrow) indicates the DNA–protein complex. NC means negative control. ns: *p* > 0.05; **: *p* < 0.01.

To clarify the regulatory relationship between LiaSR and MntR, P*_mntR_-gfp* expression was compared in 10403S, Δ*liaSR*, and CΔ*liaSR* under different pH conditions. Fluorescence results showed that at pH 4.5, pH 7 and pH 9 (Figure 4A), the fluorescence value of Δ*liaSR* was significantly higher than that of 10403S, and the fluorescence value of CΔ*liaSR* was not significantly different from that of 10403S. The above results indicated that the deletion of *liaSR* significantly increased *mntR* expression.

To further explore the mechanism of LiaSR regulating MntR, the binding ability of LiaR to P*_mntR_* was also tested. EMSA results showed that a mobility shift was observed after adding 10 μg, 8 μg, and 6 μg of LiaR protein, respectively (Figure 4B), indicating that LiaR directly bound to P*_mntR_*.

### 3.5. MntR Negatively Regulates the Expression of liaSR Through an Indirect Feedback Mechanism

**Figure 5:**
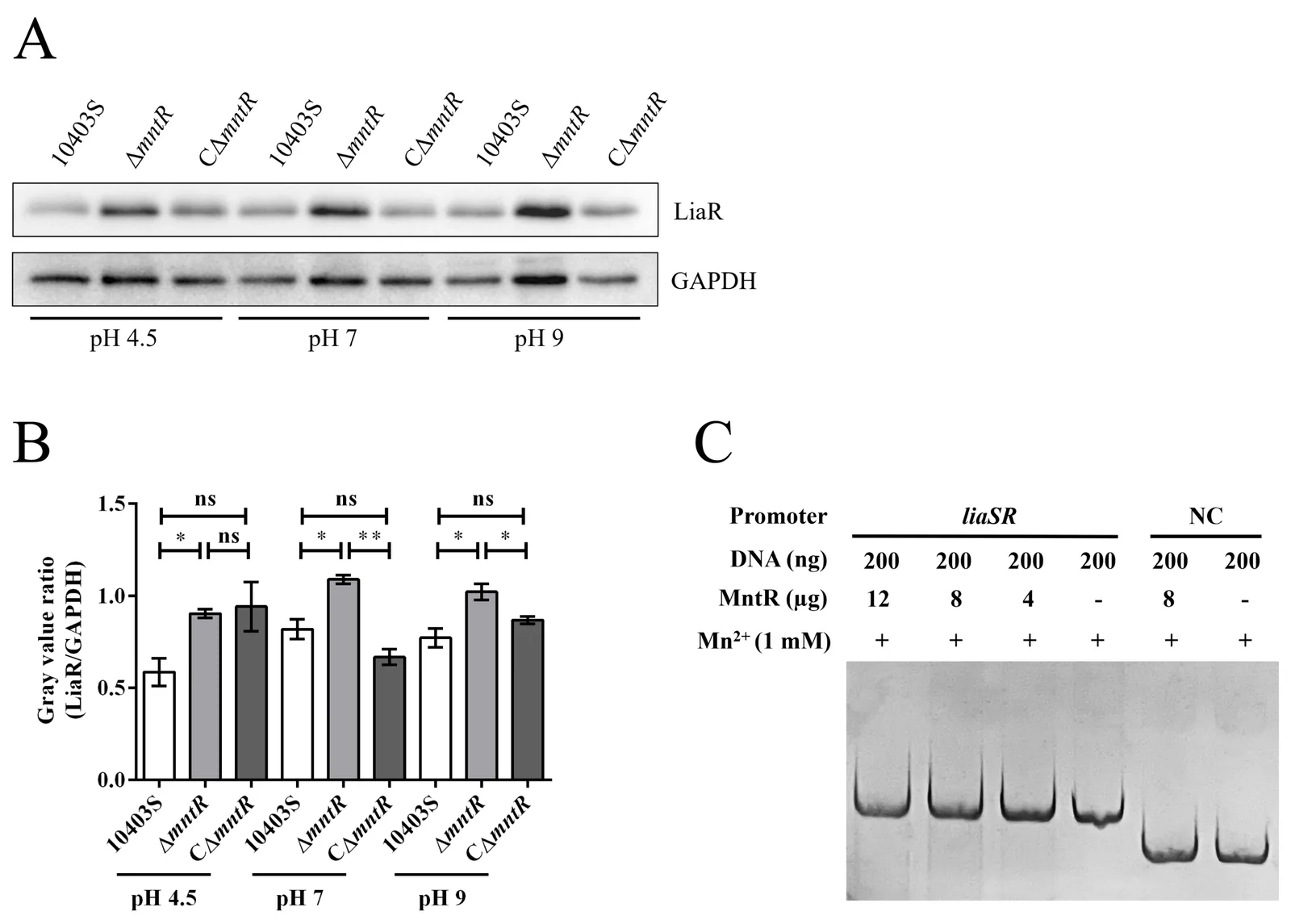
The regulation of LiaSR by MntR. (**A**) Expression of LiaR in 10403S, Δ*mntR*, and CΔ*mntR* under pH 4.5, pH 7, and pH 9 conditions. (**B**) Grayscale analysis of LiaR expression. (**C**) The electrophoretic mobility shift assay (EMSA) of MntR and P*_liaSR_*. ns: *p* > 0.05; *: *p* < 0.05; **: *p* < 0.01.

After showing that LiaSR regulates MntR expression, the effects of *mntR* deletion on LiaSR expression under different pH conditions and the binding ability of MntR to P*_liaSR_* were evaluated. Western blot results showed that at pH 4.5, the expression of LiaR in Δ*mntR* was significantly higher than that of 10403S, and the expression of LiaR in CΔ*mntR* was not significantly different from that of 10403S (Figure 5A,B). The expression of LiaR in Δ*mntR* was significantly higher than that of 10403S and CΔ*mntR* at pH 7 and pH 9, and the expression of LiaR in CΔ*mntR* was not significantly different from that of 10403S (Figure 5A,B), indicating that MntR negatively regulated LiaSR expression. However, the EMSA showed that MntR could not directly bind to P*_liaSR_* (Figure 5C), indicating that the regulation is indirect.

### 3.6. MntR Plays an Important Regulatory Role in the LiaSR-MntR-gadT2/gadD2 Regulatory Axis

**Figure 6:**
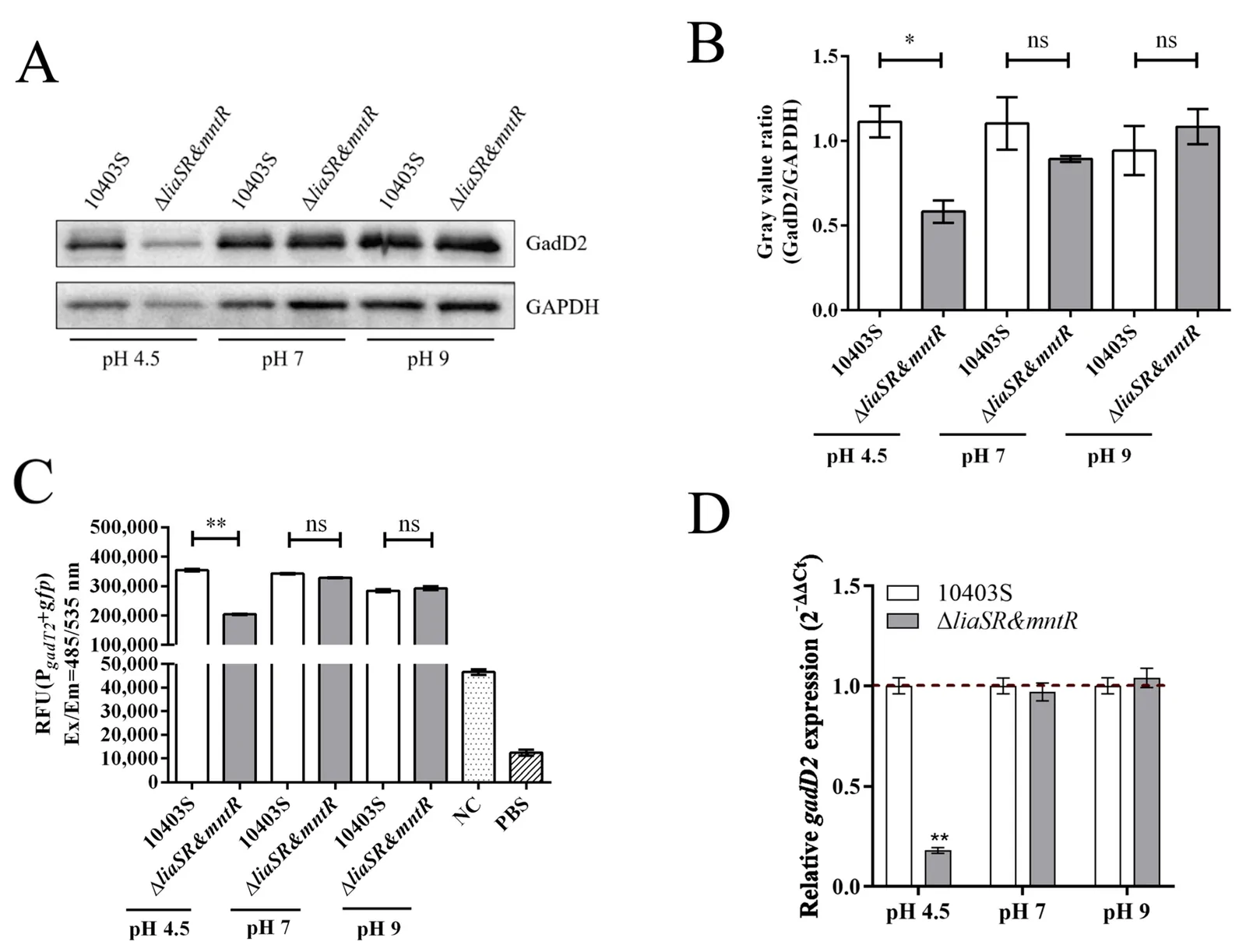
The effects of the deletion of *liaSR*/*mntR* on the transcription and expression of *gadT2*/*gadD2* under different pH conditions. (**A**) Expression of GadD2 in 10403S and Δ*liaSR*/*mntR* under pH 4.5, pH 7, and pH 9 conditions. (**B**) Grayscale analysis of GadD2 expression. (**C**) Expression of P*_gadT2_*-*gfp* in 10403S and Δ*liaSR*/*mntR* under pH 4.5, pH 7, and pH 9 conditions. (**D**) Relative *gadD2* transcript abundance determined by RT-qPCR. The expression level of 10403S under each pH condition was set to 1. Data are presented as the mean ± SD from three independent biological replicates. ns: *p* > 0.05; *: *p* < 0.05; **: *p* < 0.01.

**Figure 7:**
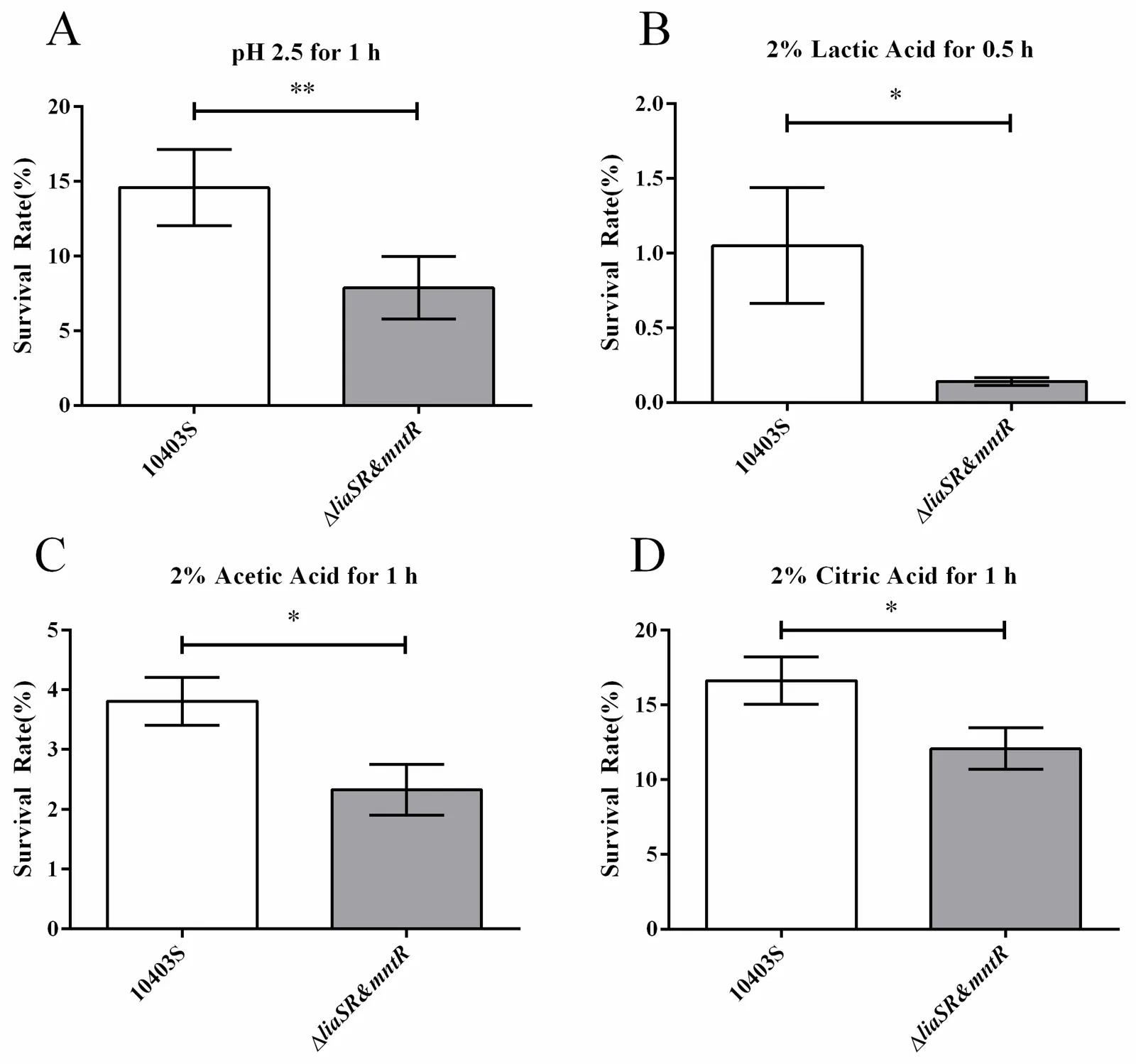
Survival assays of 10403S and Δ*liaSR*/*mntR* under various acidic conditions: (**A**) pH 2.5 HCl for 1 h, (**B**) 2% lactic acid for 0.5 h, (**C**) 2% acetic acid for 1 h, (**D**) 2% citric acid for 1 h. The mean ± SD of three independent experiments is shown in the data. *: *p* < 0.05; **: *p* < 0.01.

To further elucidate the mechanism by which LiaSR regulates *gadT2*/*gadD2* through MntR, the Δ*liaSR*/*mntR* double-deletion strain was constructed, and *gadT2*/*gadD2* expression and acid resistance were compared between 10403S and the double-deletion strain under different pH conditions. Fluorescence results showed that under pH 4.5 conditions, the fluorescence value of Δ*liaSR*/*mntR* was significantly lower than that of 10403S, whereas under pH 7 and pH 9 conditions, there was no significant difference in fluorescence values between the two strains (Figure 6C). Western blot results were consistent with the fluorescence results, showing that at pH 4.5, the expression of Δ*liaSR*/*mntR* was significantly lower than that of 10403S, while no significant differences were observed under pH 7 and pH 9 conditions (Figure 6A,B). RT-qPCR analysis further showed that *gadD2* transcript abundance in Δ*liaSR*/*mntR* was significantly lower than that in 10403S at pH 4.5, whereas no significant differences were observed at pH 7 or 9 (Figure 6D). Stress survival assays indicated that after treatment with PBS at pH 2.5 for 1 h (Figure 7A), 2% lactic acid in BHI for 0.5 h (Figure 7B), 2% acetic acid in BHI for 1 h (Figure 7C), or 2% citric acid in BHI for 1 h (Figure 7D), the survival rate of Δ*liaSR*/*mntR* was significantly lower than that of 10403S. These results are consistent with those obtained from the single deletion of *mntR* (Figure 2 and Figure 3) and in contrast to the results obtained from the single deletion of *liaSR* [23], indicating that MntR plays an important regulatory role in the LiaSR-MntR-*gadT2*/*gadD2* regulatory axis.

## 4. Discussion

*L. monocytogenes* has multiple acid-resistance systems that help it survive in acidic environments [6]. The GAD system is important for acid resistance in *L. monocytogenes*, the *gadT2*/*gadD2* component has an important role under strongly acidic conditions, and acid resistance is related to *gadT2*/*gadD2* expression [17]. The factors that affect the expression of *gadT2*/*gadD2* include gastric juice, anaerobic environment and rich nutrient medium [32,33,34]. Previous work confirmed that *gadT2*/*gadD2* responds to pH changes, and found that GadR4 inhibits the expression of *gadT2*/*gadD2* [15]. Although many factors affecting the expression of *gadT2*/*gadD2* are known, the regulatory mechanism of its acid resistance is still unclear.

Two-component systems (TCSs) play a major role in signal transduction in bacteria, transmitting extracellular environmental signals into the cell and thereby enabling corresponding transcriptional regulation [18]. In *L. monocytogenes*, TCSs have been reported to be involved in regulating various stress responses [21]. A previous TCS screen found that deletion of *liaSR* significantly increased the expression of *gadT2*/*gadD2* [23], but no direct binding of LiaR to the promoter of *gadT2*/*gadD2* was observed, indicating that LiaSR regulates *gadT2*/*gadD2* indirectly, and a transcription factor may be involved in this process.

In *L. monocytogenes*, apart from the previously reported GadR4 [15], no other transcription factors have been found to be associated with *gadT2*/*gadD2*. In this study, deletion mutants were constructed for 11 transcription factors that had not previously been linked to *gadT2*/*gadD2* regulation in *L. monocytogenes* and were screened using the *gfp* reporter system (Figure S1). By using a *gfp* reporter system, among these 11 transcription factors, only *mntR* deletion significantly reduced the expression of *gadT2*/*gadD2* (Figure 1). Although the fluorescence curves showed that after 8 h, the fluorescence values of Δ*lmo0575*, Δ*lmo0902* and Δ*glnR* were higher than the value of 10403S, the growth curves showed that their OD_600nm_ values were also higher than the value of 10403S (Figure 1D–F), suggesting that differences in bacterial density may have affected the fluorescence signals. Only the fluorescence value of Δ*mntR* was significantly lower than that of 10403S, but the former’s OD_600nm_ value was higher than the latter’s value (Figure 1J), which further indicates that MntR is associated with *gadT2*/*gadD2* expression. MntR belongs to the DtxR/MntR metal sensor family, and it requires binding to Mn^2+^ to function [35]. MntR mainly participates in regulating Mn^2+^ transport and maintaining Mn^2+^ homeostasis [36]. In *Bacillus subtilis*, MntR can both inhibit the expression of two Mn^2+^ uptake systems, MntH and MntABCD, and activate the expression of two Mn^2+^ efflux systems, MneP and MneS [36]. MntR also participates in regulating bacterial oxidative stress response. It has been shown that in *Streptococcus oligofermentans*, MntR reduces the uptake of Mn^2+^ by inhibiting MntABC, and Mn^2+^ helps to resist H_2_O_2_ oxidative stress [37]. However, the relationship between MntR and acid resistance has not been reported. After identifying MntR as a regulator of *gadT2*/*gadD2*, this study further examined the acid-resistant function of MntR. The results showed that *mntR* deletion significantly weakened the resistance of *L. monocytogenes* 10403S to organic and inorganic acids (Figure 3), indicating that MntR affected the acid resistance of *L. monocytogenes* by positively regulating the expression of *gadT2*/*gadD2*.

In *Streptococcus mutans*, there is an Mn^2+^-dependent regulator, SloR, that is similar to MntR, and a two-component system, GcrR, that is similar to LiaSR. Studies have shown that SloR can regulate the expression of GcrR and affect the acid tolerance of *Streptococcus mutans* [38]. A similar relationship may also exist between MntR and LiaSR in *L. monocytogenes*. This study first examined the regulation of MntR by LiaSR and found that LiaSR could inhibit the expression of MntR, and LiaR directly bound to P*_mntR_* (Figure 4). The results also showed that MntR could in turn inhibit the expression of LiaSR (Figure 5). Additionally, the Δ*liaSR*/*mntR* double-deletion strain was constructed (Figure S2), and *gadT2*/*gadD2* expression and acid resistance were evaluated (Figure 6 and Figure 7). The results showed that *liaSR*/*mntR* deletion significantly downregulated the expression of *gadT2*/*gadD2* under acidic conditions and weakened the acid resistance of *L. monocytogenes*. This is consistent with the results obtained from the single deletion of *mntR* (Figure 2 and Figure 3), but in contrast to the results obtained from the single deletion of *liaSR* [23]. This suggests that, in *L. monocytogenes* 10403S, the transcription factor MntR has a stronger regulatory effect on the GAD system and acid resistance compared to the two-component system LiaSR. This is understandable, as the role of a two-component system is to sense changes in the external environment and relay information to downstream target genes; however, the actual regulatory effect may be mediated by other transcription factors. Furthermore, it is possible that MntR may also be involved in regulating other acid-resistant systems in *L. monocytogenes*, because differences in the expression of *gadT2*/*gadD2* after the *liaSR*/*mntR* double deletion were only observed under acidic conditions, while no differences were noted under neutral or alkaline conditions. This indicates that under neutral and alkaline conditions, the negative regulation of *gadT2*/*gadD2* by LiaSR may balance the positive regulation by MntR, resulting in no change in *gadT2*/*gadD2* expression upon double deletion. However, under acidic conditions, other acid-resistant systems in *L. monocytogenes* are also activated, which may necessitate a higher expression level or stronger function of MntR for regulatory participation. This disruption in the balance between MntR and LiaSR regulation of *gadT2*/*gadD2* leads to the downregulation of *gadT2*/*gadD2* expression in the double-deletion context.

**Figure 8:**
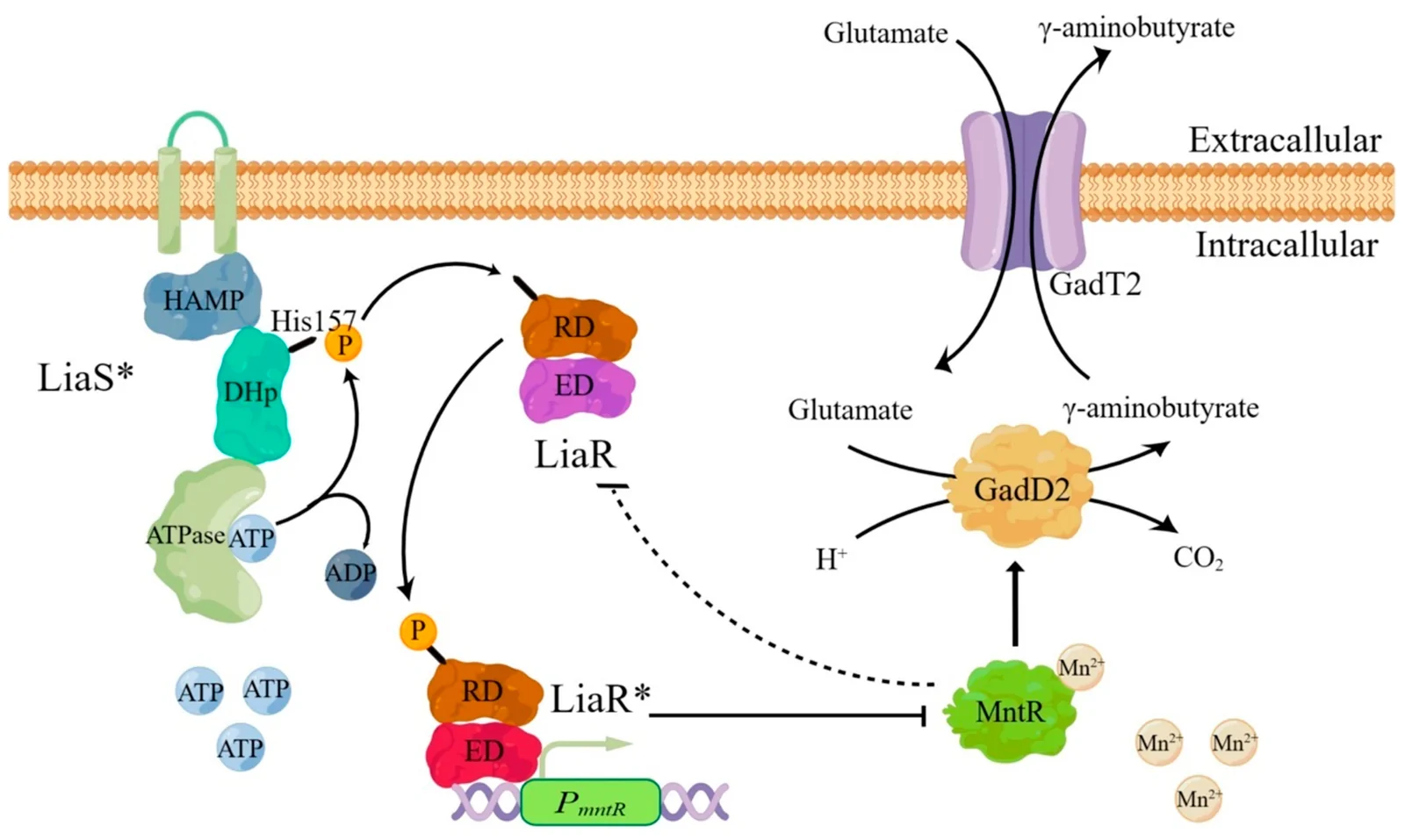
Schematic diagram of LiaSR regulating *gadT2*/*gadD2* through MntR (LiaS* denotes the autophosphorylated form of LiaS, whereas LiaR* denotes the activated form of LiaR). The figure was created with Figdraw (available online: www.figdraw.com).

Based on these findings and previous reports [19], a regulatory model was proposed in which LiaSR regulates *gadT2*/*gadD2* through MntR (Figure 8). LiaS may sense extracellular pH changes, and its kinase domain (DHp) may undergo autophosphorylation (LiaS*). Activated LiaS may transfer the phosphate group to the aspartate residue of the receiver domain (RD) of LiaR, phosphorylating the aspartate residue and activating LiaR (LiaR*). The binding domain (ED) of the activated LiaR* binds to the promoter (P*_mntR_*) of *mntR*, inhibiting the expression of MntR, and MntR may negatively regulate LiaSR expression through feedback. The manganese-activated MntR positively regulates the expression of *gadT2*/*gadD2* by binding to their promoter (P*_gadT2_*), thereby mediating the acid resistance of *L. monocytogenes* 10403S. Under acidic conditions, reduced LiaSR expression may relieve repression of *mntR*, allowing increased MntR expression and enhanced *gadT2*/*gadD2* expression (Figure 2, Figure 4 and Figure 5), thereby contributing to tolerance of *L. monocytogenes* 10403S at pH 2.5; following GAD-mediated proton consumption, feedback regulation involving MntR may increase LiaSR expression, which could in turn reduce *gadT2*/*gadD2* expression and modulate the acid-stress response.

This study has several limitations. The dependence of *L. monocytogenes* MntR activity on Mn^2+^ was not directly examined, the factor(s) mediating the indirect feedback regulation of LiaSR by MntR remain unknown, and the phosphorylation states of LiaS and LiaR were not measured. Further studies combining metal-dependence assays, ChIP-qPCR or genome-wide binding analysis, and genetic identification of the intermediate regulator will be required to refine the proposed regulatory model.

In summary, this study identified a transcription factor, MntR, that mediates the acid resistance of *L. monocytogenes* by positively regulating *gadT2*/*gadD2*. The two-component system LiaSR negatively regulates MntR, and MntR can negatively regulate LiaSR expression through an indirect feedback mechanism. These findings expand the regulatory network of the GAD system and provide a basis for the prevention and control of *L. monocytogenes*.

## 5. Conclusions

This study identified MntR as a positive regulator of *gadT2*/*gadD2* expression and acid resistance in *L. monocytogenes* 10403S. Deletion of *mntR* reduced *gadT2*/*gadD2* expression and decreased survival under inorganic and organic acid stresses. LiaSR negatively regulated *mntR* expression, and LiaR directly bound to P*_mntR_*. MntR also negatively regulated *liaSR* expression through an indirect mechanism. Together, these results support a LiaSR-MntR-*gadT2*/*gadD2* regulatory pathway and provide a basis for further studies on acid-stress adaptation in *L. monocytogenes*. Further work is needed to determine the Mn^2+^ dependence of this regulation and to identify the intermediate factor involved in MntR-mediated feedback regulation of LiaSR.

## Data Availability

The original contributions presented in this study are included in the article/Supplementary Materials. Further inquiries can be directed to the corresponding authors.

## Supplementary Materials

The following supporting information can be downloaded at: https://www.mdpi.com/article/10.3390/microorganisms14081765/s1, Table S1: Primers used in this study; Figure S1: Construction of transcription factor deletion strain of *L. monocytogenes* 10403S; Figure S2: Construction of *liaSR*/*mntR* deletion strain of *L. monocytogenes* 10403S.
