# Supplementary material for: MntR Mediates LiaSR-Regulated gadT2/gadD2 Expression and Acid Resistance in Listeria monocytogenes 10403S
Source: Microorganisms. 2026 Aug 11;14(8):1765. doi: 10.3390/microorganisms14081765 (PMC13515600; doi:10.3390/microorganisms14081765)
Supplement: Supplementary file 1 [file microorganisms-14-01765-s001.zip › Table S1.pdf]

Table S1 Primers used in this study

Primers	Sequences(5'-3')
Δ*lmo0382*-a	CATGCAGGTCGACTCTAGAGTCCTAAAGATGCTCAAGTTCGA
Δ*lmo0382*-b	TTTGCCAAAGGATGAATTTAAATTTATGTGATGAAGGAAGCCCC
Δ*lmo0382*-c	TTTAAATTCATCCTTTGGCAAA
Δ*lmo0382*-d	ATTCGAGCTCGGTACCCGGGTGCGAATTCGACATTTTCTATT
Δ*lmo0382*-e	GCGCACCGATCACTACATTG
Δ*lmo0488*-a	CATGCAGGTCGACTCTAGAGCGCAGAAGGAAAACCAAATA
Δ*lmo0488*-b	CATTCCGAGGAGGTTAACCGAAAAGGAGCCAACCAAATCG
Δ*lmo0488*-c	CGGTTAACCTCCTCGGAATG
Δ*lmo0488*-d	ATTCGAGCTCGGTACCCGGGTGCCACCTTTTTTAGATGGA
Δ*lmo0488*-e	TTAGTTTGACGATACGGGCG
Δ*lmo0547*-a	CATGCAGGTCGACTCTAGAGAAACGAGTTGTTTTTTAGCA
Δ*lmo0547*-b	GATGTAAATGGAGGTAAGTAGACCTTACTATAAAATACAAAACCC
Δ*lmo0547*-c	TCTACTTACCTCCATTTACATC
Δ*lmo0547*-d	ATTCGAGCTCGGTACCCGGGATCTTCTAAAATGGAATAACGT
Δ*lmo0547*-e	GAGTTCTCCCAAATATTTCATG
Δ*lmo0575*-a	CATGCAGGTCGACTCTAGAGAGGATTTAGCGATAATTAAA
Δ*lmo0575*-b	TTTTTCTAGCTTCTTTTTATTGGAAGTTCACCTCTTTACAC
Δ*lmo0575*-c	AATAAAAAGAAGCTAGAAAAA
Δ*lmo0575*-d	ATTCGAGCTCGGTACCCGGGTCCTTTGATAATACTATTTTTT
Δ*lmo0575*-e	ATACTAGCAACTGTTTGGCG
Δ*lmo0902*-a	CATGCAGGTCGACTCTAGAGTGGACTCCCATTGCCACATA
Δ*lmo0902*-b	TTACGGGCATGCTGACTTTATTTTCGATCGCTCCTTCACTTG
Δ*lmo0902*-c	AATAAAGTCAGCATGCCCGTAA
Δ*lmo0902*-d	ATTCGAGCTCGGTACCCGGGAACCGCCGCAACAGTTATTT
Δ*lmo0902*-e	GGGGCAAAAGGAATGTTTATC
Δ*glnR*-a	CATGCAGGTCGACTCTAGAGGAAATGTATCAAGGTTTCTT
Δ*glnR*-b	TAAATACCTCTTCTTGTCTTTCCCTCAAAACATAATTATTT
Δ*glnR*-c	AAAGACAAGAAGAGGTATTTA
Δ*glnR*-d	ATTCGAGCTCGGTACCCGGGACGATTTTCATCTAATTTAAA
Δ*glnR*-e	GACTAATTTAAATGTTTGGAT
Δ*lmo1411*-a	CATGCAGGTCGACTCTAGAGGGGGAGTGAATGTACATGAAG
Δ*lmo1411*-b	ATCTGACTATGACAAATCTAACGCTCGCTCCAATCATAAAGTTTC
Δ*lmo1411*-c	GCGTTAGATTTGTCATAGTCAGAT
Δ*lmo1411*-d	ATTCGAGCTCGGTACCCGGGCCTATCCCAAGCATTCATTG
Δ*lmo1411*-e	GTGTTAGTTTGTTAATCAGCGG
Δ*lmo1716*-a	CATGCAGGTCGACTCTAGAGTATTGATAGTTTTACTTTTCC
Δ*lmo1716*-b	TCTGTAAAATAAGAGATTAATGAATCTAATCACTTCCTAAAA
Δ*lmo1716*-c	CATTAATCTCTTATTTTACAGA
Δ*lmo1716*-d	ATTCGAGCTCGGTACCCGGGAGTGGAAATTACTGTTCAAG
Δ*lmo1716*-e	GAAAATATCCTATCTTACCA
Δ*gltC*-a	CATGCAGGTCGACTCTAGAGCTTTGTTACATCAGTTGGTAA
Δ*gltC*-b	GCATTTTCTGAAAATAATTAACTCGAAATGAGATTGTTTTA
Δ*gltC*-c	TTAATTATTTTCAGAAAATGC
Δ*gltC*-d	ATTCGAGCTCGGTACCCGGGCGATGAAACAGTTAGAAGATA
Δ*gltC*-e	ATACGGAATCTTAGTTCAATT
Δ*mntR*-a	CATGCAGGTCGACTCTAGAGAATAAATGCCGATAAAGCATT
Δ*mntR*-b	AATTTAAGTTGGAGGGTATAGCTCGGTAATCCAAGGTTGAGAT
Δ*mntR*-c	AGCTATACCCTCCAACTTAAATT
Δ*mntR*-d	ATTCGAGCTCGGTACCCGGGCGGTTTTTAAGTCCGTGTCT
Δ*mntR*-e	AATTCAGCCTCATGATTTGTC
Δ*lmo2233*-a	CATGCAGGTCGACTCTAGAGACACGATATCCAGTTATTGATG
Δ*lmo2233*-b	AACTTTTTAGTTAGGAGAGAAGCAAAAGTAGCTACCCTATGAGAG
Δ*lmo2233*-c	GCTTCTCTCCTAACTAAAAAGTT
Δ*lmo2233*-d	ATTCGAGCTCGGTACCCGGGGTGGAAAGTTGTTTGCTCTT
Δ*lmo2233*-e	TCACAAATAAGTTGCGCGTTA
CΔ*mntR*-F	GAGTGAAACCCATGGAAAAGATGCCAACACCTAGTATGGAAG
CΔ*mntR*-R	CGAATTCCTGCAGCCCGGGGTTATTCCTTTGTTTCTGGTTTCG
P_{mntR}-fwd	CCGGAATTCCCGGGGATCCGCCGTGTCTAATTGGCAACTG
P_{mntR}-rev	TGAAAAGTTCTTCTCCTTTACTCATAGCTATACCCTCCAACTTAAATTCT
gfp-fwd	ATGAGTAAAGGAGAAGAACTTTTCA
gfp-rev	ATATACTAACCCTTTTTTTGTTATTTGTATAGTTCATCCATGCC
gadD2-fwd	CCTTTGGAAAGATGAAAGCTAC
gadD2-rev	TGTAGTATTGACCGATGATGTG
gyrB-fwd	AGACGCTATTGATGCCGATGA
gyrB-rev	GTATTGCGCGTTGTCTTCGA
